# Metabolic Heterogeneity and Niche Rewiring in Plasma Cells are Associated with Progression from MGUS to Multiple Myeloma

**DOI:** 10.21203/rs.3.rs-8795835/v1

**Published:** 2026-02-17

**Authors:** Axel Walch, Guoxing Zhang, Na Sun, Yogesh Chawla, Dragan Jevremovic, Hitosugi Taro, Shaji Kumar, Wilson Gonsalves

**Affiliations:** helmholtz zentrum münchen; helmholtz zentrum münchen; helmholtz zentrum mü; Mayo Clinic; Mayo Clinic; Mayo Clinic; Mayo clinic; Mayo Clinic

## Abstract

Progression from monoclonal gammopathy of undetermined significance (MGUS) to multiple myeloma (MM) is driven by coordinated metabolic reprogramming within clonal plasma cells and the bone marrow microenvironment. We applied high-resolution MALDI–FT-ICR mass spectrometry imaging (MSI) to archived FFPE bone-marrow biopsies, integrated with matched bone marrow plasma metabolomics, to map spatial and systemic metabolic alterations. Spatial clustering delineated plasma-cell–rich niches, while Hill-based diversity and β-diversity metrics quantified intra- and inter-compartment heterogeneity. MM niches exhibited elevated 3-hydroxykynurenine, rewired tryptophan–kynurenine flux, and increased nucleotide and bioactive lipid metabolism associated with proliferation. Notably, some MGUS-like samples displayed MM-like metabolic niches undetectable in bone marrow plasma alone, underscoring spatial heterogeneity. Cross-compartment integration revealed conserved metabolic signatures and systemic redistribution of key metabolites, consistent with ecological reorganization and niche divergence during progression. These findings establish spatial metabolomics of biopsies as a framework to dissect intramedullary metabolic heterogeneity and enable metabolite-based risk stratification in plasma-cell disorders.

## Introduction

Multiple myeloma (MM) represents the malignant phase of a clonal plasma-cell continuum, invariably preceded by monoclonal gammopathy of undetermined significance (MGUS) and often an intermediate smouldering phase^1^. Reliable biomarkers capable of predicting MGUS progression remain unavailable^2^. Given the complexity of the bone marrow microenvironment, disease evolution likely involves both clonal plasma cells and surrounding microenvironmental components^3^. Characterizing metabolic profiles of clonal plasma cells within their bone marrow niche may illuminate mechanisms of disease progression.

Metabolic reprogramming is a hallmark of cancer, enabling cells to meet increased biosynthetic and energetic demands associated with rapid proliferation^4^. In MM, pathways related to fatty acid, glucose, and amino acid metabolism are notably upregulated to facilitate nucleotide and protein synthesis, as well as ATP production^5,6^. However, conventional bulk metabolomics analyses typically use homogenized tissue or plasma samples, lacking spatial resolution and thus failing to localize metabolic signals to specific anatomical sites^7,8^.

Recent advances in spatial metabolomics, particularly MSI, now enable in situ visualization of metabolite distributions within tissue sections^9^. Unlike bulk approaches, spatial metabolomics can directly map metabolites to tumor, stromal, and immune cells within their native microenvironment, revealing spatial heterogeneity^10^.

Few studies have directly investigated metabolic changes associated with MGUS-to-MM progression using bone marrow or plasma metabolomics. Prior work identified substantial metabolic differences between MGUS and MM patients in bone marrow plasma, including decreased branched-chain amino acids and elevated kynurenine-pathway metabolism leading to increased 3-hydroxykynurenine (3-HK) production in MM^11^. Additionally, serum markers linked to tryptophan metabolism correlate with MM progression^12^. However, previous studies have primarily focused on bulk-level differences without integrating tissue-localized and systemic metabolomics or achieving sub-tissue resolution^13^. Notably, spatial metabolomics using high-mass-resolution MALDI–FT-ICR-MSI has not previously been applied systematically to diagnostic FFPE bone marrow biopsies, representing a methodological and clinical innovation that allows interrogation of plasma cell metabolism within its native niche context.

Here, we apply high-mass-resolution MALDI-FT-ICR-MSI systematically to archival FFPE bone marrow biopsies, integrated with matched extracellular bone marrow plasma metabolomics, to map spatially resolved metabolic profiles. By quantifying intra- and inter-compartment heterogeneity and identifying conserved metabolic signatures across plasma cells and microenvironmental niches, this study provides a holistic framework for understanding metabolic evolution during MGUS-to-MM progression. These insights establish a foundation for metabolite-based risk stratification and the identification of targetable metabolic pathways in pre-malignant plasma cell disorders.

## Methods

### Patient Cohort

This retrospective observational study was approved by the Mayo Clinic Institutional Review Board (IRB#: 15–005140) and conducted in accordance with the Declaration of Helsinki. Clinical residual bone marrow plasma and trephine biopsy samples were obtained from consecutive patients with newly diagnosed multiple myeloma (NDMM) or MGUS-like bone marrows undergoing routine diagnostic evaluation, with informed consent for research use. Diagnoses were confirmed according to International Myeloma Working Group (IMWG) criteria. Relevant clinical and laboratory parameters were collected and are summarized in Table 1. Additional cohort details are provided in the Supplemental Methods.

### LC-MS assessment of bone marrow plasma

Bone marrow plasma samples were subjected to global untargeted metabolomic profiling by Metabolon, Inc. using ultrahigh-performance liquid chromatography–tandem mass spectrometry (UPLC–MS/MS), as previously described. Analyses were performed in both positive and negative ion modes, and metabolites were identified by comparison to authenticated reference standards.

### High-mass-resolution MALDI-FT-ICR-MSI analysis

MALDI-FT-ICR-MSI was performed on archived FFPE bone-marrow biopsy sections from Mayo Clinic. Imaging was conducted in negative ion mode with a spatial resolution of 50 μm over a mass range of *m/z* 75–1000. Following MSI acquisition, sections were subjected to H&E staining for histological annotation.

### Data Acquisition and Processing

MALDI-MSI data were processed and annotated using established software pipelines, including spectral normalization and accurate-mass-based metabolite annotation against curated databases.

### Statistical analysis

Statistical analyses were performed using GraphPad Prism (v9.4.1), R (v4.4.3), and Python (v3.13). Twogroup comparisons were conducted using two-sided Student’s t-tests, with Welch’s correction applied when variances were unequal; nonparametric Mann–Whitney U tests were used for non-normally distributed data. Correlations between continuous variables were assessed using two-tailed Pearson or Spearman correlation coefficients, as appropriate. Unsupervised multivariate analyses were applied to explore metabolic patterns and group differences. All tests were two-sided, with significance defined as P < 0.05. Additional details about methods and analyses are provided in the Supplemental Methods.

## Results

### Study Design

**Figure 1:**
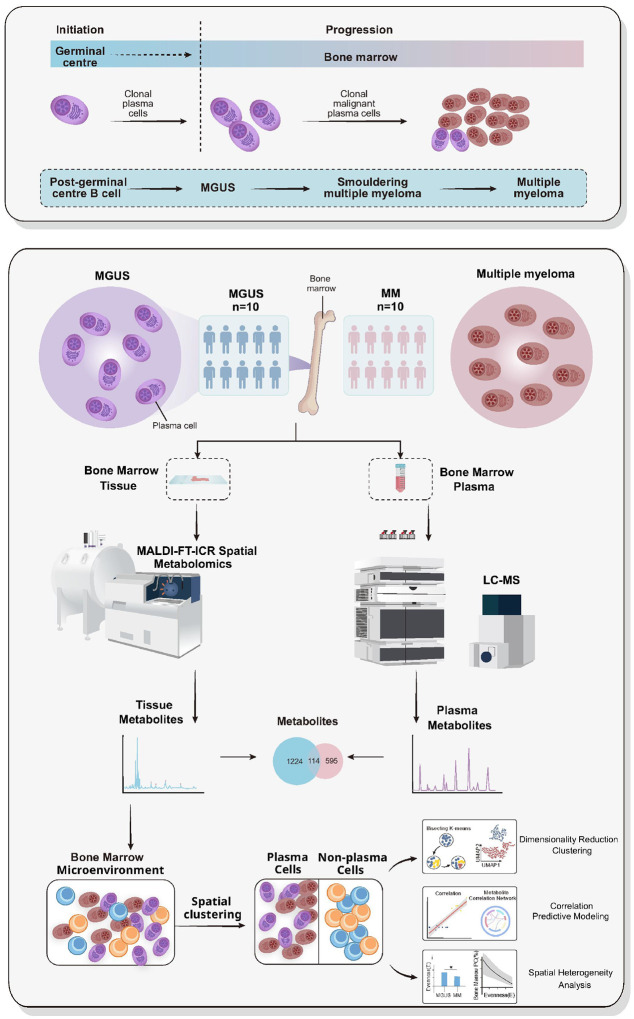
Workflow. Study workflow and MALDI-FT-ICR–MSI pipeline. **Top panel** (disease course): Schematic representation of plasma-cell tumorigenesis from post-germinal-centre B cells through MGUS and smouldering multiple myeloma to overt MM. **Bottom panel** (sample acquisition, spatial metabolomics, and computational analysis): Bone-marrow tissue and matched plasma were collected from patients with MGUS (n = 10) and MM (n = 10). FFPE bone-marrow sections were analysed by high-mass-resolution MALDI–FT-ICR mass-spectrometry imaging (MSI), and matched plasma was profiled by LC–MS. Integration of both matrices yielded a shared metabolite set for cross-compartment comparison. Spatial clustering of MSI data delineated bone-marrow microenvironmental compartments, distinguishing plasma-cell–rich regions (purple, brown) from non–plasma-cell immune regions (orange, blue). These spatially defined compartments formed the basis for downstream analyses, including dimensionality reduction and clustering, spatial heterogeneity assessment, and correlation-based or predictive modelling.

We analyzed paired bone marrow plasma samples and formalin-fixed paraffin-embedded (FFPE) bone marrow core biopsies from 10 patients with symptomatic, newly diagnosed multiple myeloma (NDMM) and 10 patients with MGUS-like bone marrows(Fig. 1). The MGUS-like cohort, hereby labelled as “MGUS” moving forward, was defined by bone marrow biopsies showing less than 10% clonal plasma cells and included individuals with true MGUS (n = 7) as well as those with solitary bone plasmacytoma with minimal marrow involvement (SBPmm; n = 3). Core biopsies from SBPmm patients were intentionally included because their histologic appearance and degree of clonal plasma cell infiltration are indistinguishable from true MGUS marrows, yet their risk of progression to MM is substantially higher. This design allowed us to assess whether metabolomic profiling could identify metabolic features in MGUS-like marrows, particularly those from SBPmm, that are biologically more similar to NDMM. The clinical characteristics of these patients are listed in Table 1.

#### Metabolomics-Driven Spatial Clustering Reveals Metabolic Heterogeneity of Bone Marrow Plasma Cells in MGUS and Multiple Myeloma

To interrogate metabolic remodeling where myeloma emerges, we first established a tissue-centric map by segmenting bone-marrow sections into plasma-cell–rich niches using MSI.

**Figure 2:**
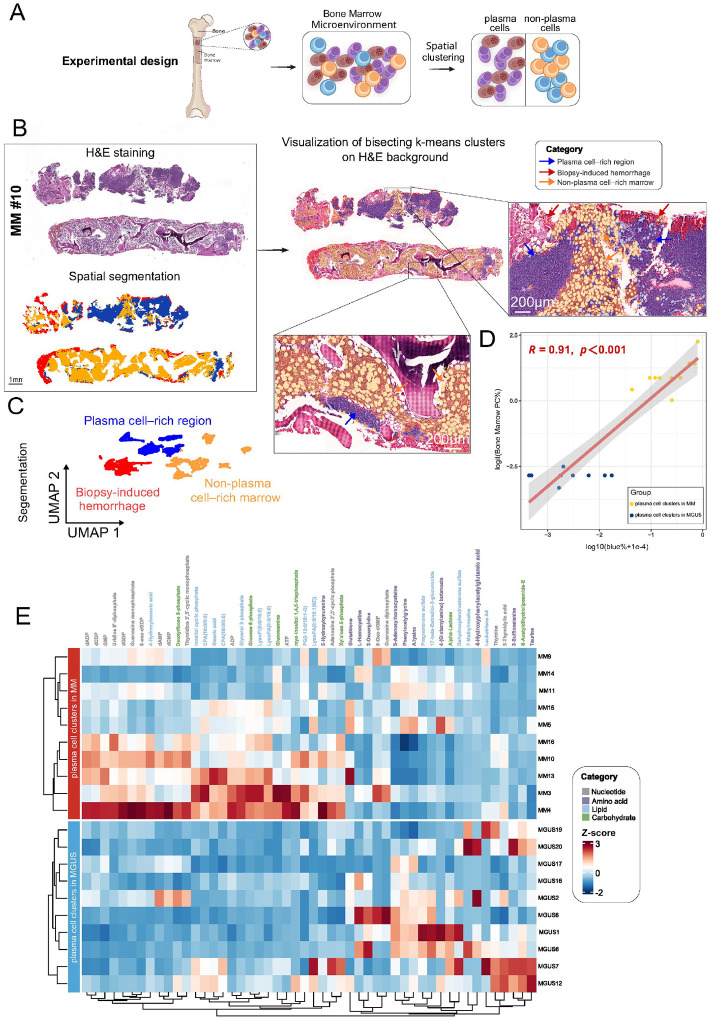
Metabolite-based clustering and segmentation delineate plasma cell–rich regionsin FFPE bone marrow samples from MGUS and MM patients. **A.** Experimental design illustrating the workflow for spatial clustering of plasma cells using metabolomic data combined with UMAP dimensionality reduction and clustering analysis. **B.** Representative MM case (#10). Top left: H&E-stained bone-marrow section. Bottom left: bisecting k-means segmentation. Middle, bisecting k-means segmentation overlaid on the H&E background. Right, zoomed insets highlight that the segmentation resolves three histologically interpretable compartments: plasma cell–rich region (blue), biopsy-induced hemorrhage (red), and non-plasma cell–rich marrow(orange). Scale bars as indicated (1 mm for whole section; 200 μm for insets). **C.** UMAP embedding of all MSI spectra from the same MM#10 section, colored by the bisecting k-means labels. The three region types form well-separated clusters, confirming concordance between unsupervised spectral clustering and histology-guided interpretation. **D.** Pearson correlation analysis between the percentage of malignant plasma cell regions (blue areas, quantified by pixel numbers in SCiLS imaging) and the percentage of bone marrow plasma cells (PC%) across 20 patients (10 MGUS, 10 MM). To meet statistical assumptions of correlation analysis, variables were transformed (x = log10[blue% + 1e–4], y = logit[Bone marrow PC%]). Each point represents an individual patient, with MGUS cases shown in blue and MM cases in orange. The shaded area denotes the 95% confidence interval of the fitted linear regression. Pearson’s correlation coefficient (R) and p-value are indicated in the plot. Spearman’s rank correlation on the original scale (ρ = 0.89, p < 1 × 10^−6^) confirmed a robust monotonic association. **E.** Heatmap of the top 50 most variable metabolites (out of 123 high-confidence endogenous metabolites) quantified by MSI within the plasma cell–rich regions (blue cluster) delineated by bisecting k-means. Metabolites were ranked by their variance across all plasma cell–rich regions in the Z-score–normalized MSI matrix, and the 50 metabolites with the highest variance were selected to optimize clustering visualization. Rows correspond to metabolites, and columns to plasma cell–rich regions segmented from individual patients (MGUS vs MM). Values are Z-score–normalized per metabolite. The left annotation bar denotes super-pathway membership (e.g., nucleotide, amino-acid, lipid, carbohydrate, energy, vitamin, other), and the top annotation distinguishes MGUS from MM. Unsupervised hierarchical clustering separates “plasma cell clusters in MGUS” from “plasma cell clusters in MM,” highlighting pathway-level differences in malignant versus precursor niches.

Using high-resolution MALDI-FT-ICR MSI data, we performed unsupervised bisecting *k*-means clustering to partition the metabolic landscapes of 20 FFPE bone marrow specimens(Fig. 2A). This clustering method, which efficiently captures intratumoral metabolic heterogeneity in MSI datasets^14^, delineated discrete tissue regions in each sample. Notably, it segregated high-purity plasma cell–rich regions out from the remainder of the bone marrow microenvironment in both MGUS and MM samples, indicating a successful metabolic separation of plasma cells in different precursor and malignant compartments (Fig. 2B). To validate the robustness of this unsupervised segmentation across patients, we projected the data using UMAP dimensionality reduction. The UMAP embeddings demonstrated a clear bifurcation between plasma cell-rich and non-plasma cell rich metabolic profiles (Fig. 2C).

We next assessed the biological fidelity of the metabolomic clusters by comparing them to standard pathology metrics. After appropriate transformation to satisfy statistical assumptions, the fraction of pixels classified as “tumor region” by MSI in each bone marrow sample strongly correlated with the percentage of plasma cells determined by conventional morphological evaluation (Pearson R = 0.91, p < 0.001; Fig. 2D). Consistent results were obtained using Spearman’s rank correlation on the original scale (ρ = 0.89, p < 1×10^−6^).

Out of 1,338 ion features detected by MALDI-MSI in the bone marrow sections, we curated a subset of 123 high-confidence endogenous metabolites as the basis for subsequent analyses. A heatmap of these metabolites within the plasma cell–rich regions (blue cluster) revealed clear separation of MGUS and MM and pathway-level differences annotated by super-pathway categories (nucleotide, amino acid, lipid, carbohydrate) (Fig. 2E). MM plasma cell rich regions showed broad upregulation across multiple pathways, consistent with increased glycolytic, nucleotide and amino-acid metabolism in malignant states compared to those plasma cell regions in MGUS^15^. Collectively, spatial metabolomic clustering segregated plasma cell–rich regions with distinct metabolic states in MGUS and MM.

### Rewiring of tryptophan metabolism distinguishes MGUS from MM progression

**Figure 3:**
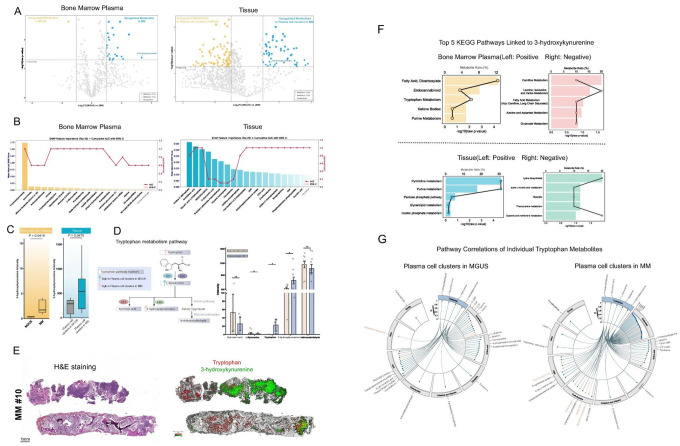
Metabolomic characterization identifies tryptophan metabolism as significantly altered in malignant plasma cells of MM. **A.** Volcano plots illustrating differential metabolite abundance between MGUS and MM malignant plasma cell regions (segmented blue regions defined in Figure 1) in bone marrow plasma (left) and FFPE bone marrow tissue samples (right). Colored dots represent significantly altered metabolites (upregulated in MGUS: yellow; upregulated in MM: blue). The tryptophan metabolite 3-hydroxykynurenine showed significant elevation in MM plasma and tissue samples. **B.** SHAP (SHapley Additive exPlanations) feature importance analysis combined with cumulative area under the curve (AUC) to rank metabolite importance for distinguishing MGUS from MM malignant plasma cell regions. Bone marrow plasma analysis (left) demonstrates 3-hydroxykynurenine as the top-ranking metabolite. Tissue analysis (right) also highlights key metabolites in metabolic distinction. SHAP values were computed using a CatBoost classifier. **C.** Box plots comparing the intensity of 3-hydroxykynurenine between MGUS and MM in bone marrow plasma (left, Welch t-test, *p* = 0.0416) and tissue samples (right, Welch t-test, *p* = 0.0470). Boxes indicate interquartile ranges, horizontal lines represent medians, and whiskers extend to the highest and lowest data points within 1.5×IQR. **D.** Tryptophan metabolic pathway highlighting five detected metabolites with their associated changes (left). Upward arrows indicate the group in which the metabolite was significantly elevated. Bar plots (right) depict intensities of these metabolites in malignant plasma cell regions, analyzed using appropriate statistical tests according to normality and variance homogeneity assumptions (Shapiro–Wilk and Levene’s tests). Three metabolites were significantly different after Benjamini–Hochberg correction for multiple comparisons: 3-hydroxykynurenine (Welch t-test, *p* = 0.047), L-kynurenine (Mann–Whitney U test, *p* = 0.021), and tryptophan (Mann–Whitney U test, *p* = 0.009). **E.** MALDI imaging mass spectrometry of MM patient #10 (FFPE tissue). H&E staining (left) and corresponding spatial distributions (right) of tryptophan (red) predominantly in pre-malignant regions, and 3-hydroxykynurenine (green) predominantly in malignant plasma cell regions. Scale bar: 1 mm. **F.** KEGG pathway over-representation for metabolites correlated with 3-HK. For bone marow plasma (top) and tissue (bottom), metabolites were split into positively (left panels; Pearson’s r > 0.30) and negatively (right panels; r < −0.30) correlated sets based on correlations with 3-HK intensity in the corresponding datasets. Each set was tested against a curated KEGG metabolite–pathway map; panels display the top five enriched pathways ranked by p value. Horizontal bars report −log10(p value), and the overlaid line with points (secondary x-axis) denotes the percentage of 3-HK–correlated metabolites annotated to each pathway (“Metabolite Ratio”, equivalent to GeneRatio). **G.** Circular correlation plots illustrating relationships between individual metabolites of the tryptophan pathway and other detected metabolites within malignant plasma cell regions of MGUS (left, “Plasma cell clusters in MGUS”) and MM (right, “ Plasma cell clusters in MM”). Bars represent correlation coefficients (|r|) of metabolites, with blue indicating positive correlations and orange indicating negative correlations. Connecting lines in the center highlight significant pairwise correlations (p < 0.05). The MGUS group contains four detected tryptophan metabolites, while the MM group contains five, reflecting differential metabolic profiles between disease states.

To identify key metabolic features associated with the malignant transformation from MGUS to MM, we performed comprehensive, untargeted metabolite profiling of FFPE bone marrow tissue using high-resolution MALDI-FT-ICR MSI and their matched bone marrow plasma by an untargeted ultra-performance liquid chromatography tandem mass spectrometer (UPLC-MS/MS). Differential analysis via volcano plots revealed numerous dysregulated compounds, notably a robust and consistent elevation of 3-HK in MM compared with MGUS across both bone marrow plasma and tissue compartments (Fig. 3A). In a SHAP-based classification model of the bone marrow plasma metabolome, 3-HK emerged as the top-ranked discriminatory feature separating MGUS from MM (Fig. 3B, **left**), whereas it was absent among the top predictors in the analogous model for tissue metabolites (Fig. 3B, **right**). Nonetheless, univariate analysis confirmed that 3-HK levels were significantly higher in MM than in MGUS in both bone marrow plasma and tissue (p < 0.05 for each; Fig. 3C).

Given the prominence of 3-HK, we next examined its position within the tryptophan degradation pathway. Five of the six key intermediates in this pathway were detectable, and three (Tryptophan, L-kynurenine and 3-HK) were significantly atered between MGUS and MM; in contrast, in the bone marrow tissue, both tryptophan and 3-HK were elevated in the plasma cells from MM comapred to those from MGUS—indicating an increased flux of extracellular tryptophan into plasma cells through the kynurenine arm during malignant progression from MGUS to MM^16^(Fig. 3D). This pattern is consistent with enhanced indoleamine-2,3-dioxygenase (IDO)-mediated catabolism of tryptophan in MM clonal cells^17^. Imaging mass spectrometry further localized this rewiring: in a representative MM section, 3-HK mapped predominantly to malignant regions consisting of plasma cell clusters, while tryptophan accumulated in adjacent areas devoid of plasma cell clusters (Fig. 3E).

To place 3-HK within a broader pathway context, we correlated 3-HK intensity with all detected metabolites in bone marrow plasma and tissue and then tested positively (r > 0.30) and negatively (r < − 0.30) correlated sets for KEGG pathway over-representation. In bone marrow plasma, positively correlated partners of 3-HK were enriched for fatty-acid/dicarboxylate, endocannabinoid and tryptophan metabolism, whereas negatively correlated partners highlighted carnitine metabolism and amino-acid catabolic routes (Fig. 3F, **top**). In tissue, positively correlated sets were dominated by pyrimidine and pentose/inositol-phosphate pathways, while negatively correlated sets included lysine and phenylalanine metabolism (Fig. 3F, **bottom**). These enrichments highlight compartment-specific pathway associations of 3-HK.

Finally, a complementary pathway-level correlation analysis across all tryptophan-pathway metabolites (Fig. 3G) showed that with progression from MGUS to MM their connectivity shifts: correlations with nucleotide and carbohydrate metabolism strengthen, whereas links to lipid metabolism weaken. This reorganization reflects broader metabolic remodeling in MM—one that favors nucleotide biosynthesis and glycolytic energy production over lipid pathways^18^.

Collectively, these results link tryptophan–kynurenine rewiring in MM plasma cells to distinctive metabolite signatures, exemplified by elevated 3-HK. Therefore, we quantified how metabolite levels couple to proliferation across disease stages.

### Metabolite–S-phase Proliferation Correlations Reveal Stage-Specific Drivers of Plasma Cell Growth

**Figure 4:**
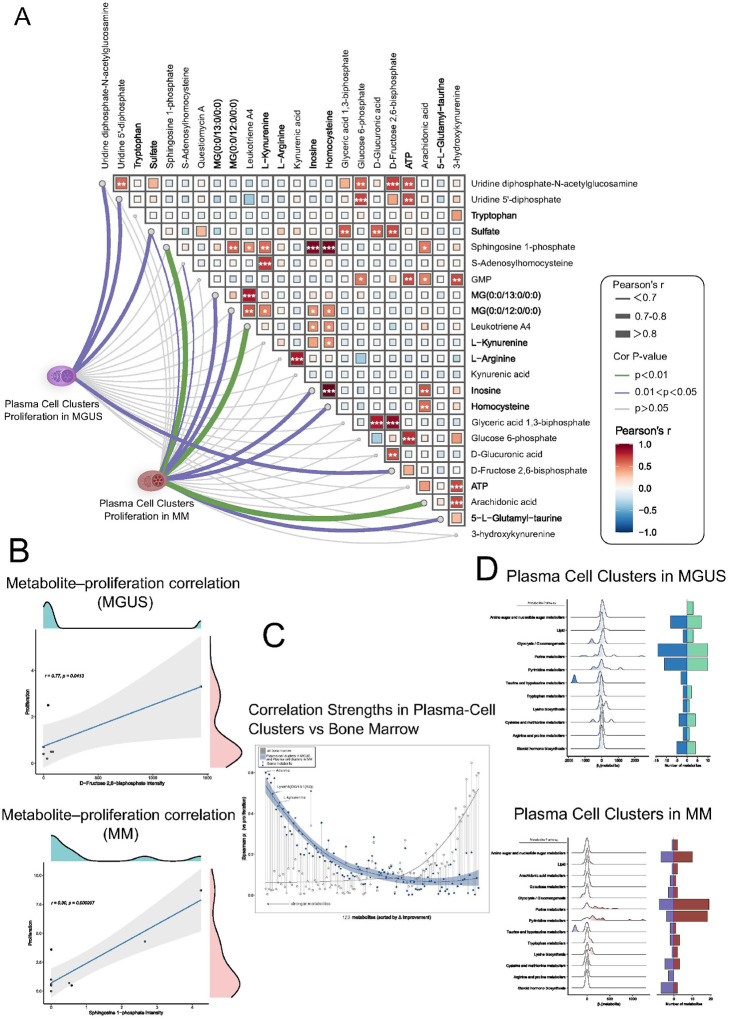
Correlation analysis between metabolite profiles and proliferation in malignant plasma cells of MGUS and MM patients. **A.** Network correlation plot illustrating associations between individual metabolites and plasma cell proliferation (left panel), alongside pairwise metabolite correlations (right panel). In the left panel, lines connect proliferation in MGUS and MM groups to specific metabolites, with line colors representing correlation significance (purple: positive correlation, *p* < 0.05; green: positive correlation, *p* < 0.01; gray: non-significant). Line thickness corresponds to correlation strength (Pearson’s r). The right triangular heatmap panel displays correlations among metabolites; tile color intensity indicates correlation strength (Pearson’s r), and statistical significance is indicated by asterisks (***p < 0.001, **p < 0.01, *p < 0.05). **B.** Scatterplots with marginal density plots illustrating representative correlations between metabolite intensities and proliferation. Upper plot: positive correlation of D-Fructose 2,6-bisphosphate intensity with proliferation in MGUS plasma cells (Pearson’s r = 0.77, *p* = 0.0493). Lower plot: positive correlation of sphingosine 1-phosphate intensity with proliferation in MM plasmacytoma cells (Pearson’s r = 0.90, *p* < 0.001). Marginal density distributions reflect data dispersion along both axes. **C.** Scatter plot comparing metabolite–proliferation correlation strengths (|Spearman ρ|) between segmented malignant plasma cell regions (blue points) and entire bone marrow regions (gray points). Each metabolite correlation is represented twice: correlations calculated within segmented malignant regions (blue) and within full bone marrow sections (gray). Metabolites are sorted along the x-axis based on the Δ|Spearman ρ|, representing the improvement in correlation strength within segmented regions compared to the full bone marrow. Lines and shaded regions represent LOESS polynomial regression fits with corresponding 95% confidence intervals. **D.** Ridgeline plots summarizing distributions of correlation coefficients (β1 from linear regression) between metabolites (grouped by metabolic pathways) and proliferation in MGUS (top) and MM (bottom). Adjacent bar charts display the number of positively (right bars) and negatively (left bars) correlated metabolites within each pathway.

We correlated all endogenous metabolites within plasma-cell rich regions in MGUS and MM bone marrow tissue with their paired proliferation score of their corresponding clonal plasma cells, revealing stage-specific drivers^19^. The triangular heat map **(right half of** Fig. 4A) showed tightly co-regulated blocks (glycolysis, nucleotide sugars, bioactive lipids) with dense within-block and sparse cross-block correlations, consistent with partly independent metabolic circuits.

Overlaying proliferation (**left half of** Fig. 4A) shows that the strength—and even the sign—of metabolite–proliferation coupling shifts with disease stage. In plasma cell clusters in MGUS, sulfate exhibits the highest positive correlation with proliferation (r = 0.79), followed by fructose-2,6-bisphosphate and two UDP-linked nucleotide sugars, hinting that redox buffering and anabolic carbohydrate flux support limited plasma-cell expansion at the premalignant MGUS stage (Fig. 4B, **top figure**). By contrast, in plasma cell clusters in MM the dominant correlates switch to the eicosanoid arachidonic acid (r = 0.91), Sphingosine 1-phosphate and Leukotriene A4—lipid mediators implicated in mitogenic signalling and NF-κB activation—together with purine derivatives such as inosine (Fig. 4B, **bottom figure**). This lipid-centric signature aligns with reports that sphingolipid and arachidonate pathways fuel aggressive myeloma proliferation and confer drug resistance^20,21^.

Comparing segmented plasma-cell regions (blue) with whole-marrow averages (grey) confirmed that spatially resolved profiling greatly amplifies the metabolite–proliferation signal (Fig. 4C); most blue points lie above their grey counterparts, with adenine showing the largest gains (Δ|ρ| > 0.25). Focusing on plasma cell-rich niches markedly strengthened the metabolite–proliferation correlations.

Finally, we observe a reversal in the behavior of nucleotide metabolism pathways between MGUS and MM. In MGUS, metabolites involved in purine and pyrimidine metabolism show predominantly negative correlations with plasma cell proliferation (**blue bars in** Fig. 4D, **top**), whereas in MM these same pathways shift to predominantly positive correlations (r**ed bars in** Fig. 4D, **bottom**). This transition suggests that, in the indolent pre-malignant MGUS state, higher proliferation in plasma cells is associated with lower steady-state levels of nucleotides – possibly reflecting consumption or a restrained de novo synthesis capacity – but in MM, proliferating plasma cells actively accumulate or maintain elevated nucleotide pools, indicating an upregulation of nucleotide biosynthesis to meet increased DNA/RNA demands^22,23^. Consistently, prior studies have noted altered nucleotide metabolism as a distinguishing feature between MGUS and MM^13^, and high expression of nucleotide biosynthetic enzymes (e.g. cytidine triphosphate synthase 1, CTPS1) correlates with aggressive MM behavior^24^. Beyond nucleotides, other pathways display stage-specific shifts as well: for example, amino sugar metabolism and glycerolipid metabolism show modest positive correlations with proliferation already in MGUS, which further intensify in MM, whereas pathways like taurine/hypotaurine metabolism (e.g. 5-L-glutamyl-taurine) remain uncorrelated in MGUS but become positively linked to proliferation in MM^25,26^.

Having identified plasma cell tissue-intrinsic metabolic drivers of proliferation, we next examined which signals are mirrored systemically by integrating matched bone marrow plasma profiles.

### A shared metabolic core links clonal plasma cells with their extracellular environment

**Figure 5:**
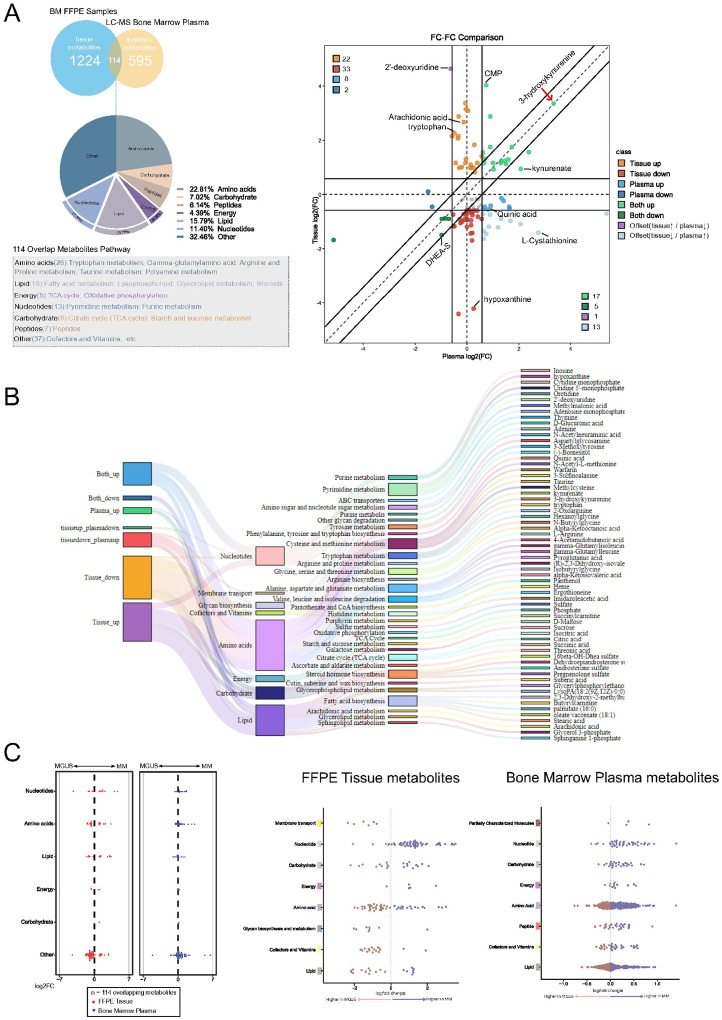
Metabolomic profiling of FFPE tissue and plasma samples by spatial metabolomics and LC-MS analysis. **A.** Venn diagram showing 114 overlapping metabolites identified from FFPE tissues and bone marrow plasma samples by MALDI and LC-MS analysis (top left). Pie chart illustrates the distribution of these overlapping metabolites among metabolic pathways (bottom left). The volcano plot (FC-FC comparison, right) visualizes the log2 fold changes (log2FC) of overlapping metabolites in bone marrow plasma (x-axis) and FFPE tissues (y-axis) between MM and MGUS samples. Metabolites are classified into eight directional groups based on log2FC thresholds (|log2FC| ≥ 0.58, corresponding to a 1.5-fold change). Colors indicate metabolite classification groups; numbers indicate the count of metabolites within each group. **B.** Sankey diagram showing the first- and second-level metabolic pathway assignments of metabolites from seven classification groups as defined in the volcano plot (excluding the Bone Marrow Plasma down group composed entirely of exogenous metabolites). **C.** Beeswarm plots illustrating log_2_ fold changes (log_2_FC) of the 114 overlapping metabolites between MGUS and MM samples across selected metabolic pathways. The left panel shows the distribution of these metabolites grouped by metabolic pathways separately for FFPE tissue (red dots) and bone marrow plasma (blue dots). The central and right panels illustrate the pathway-level distribution of all detected metabolites from FFPE tissue samples and bone marrow plasma samples, respectively. Each dot represents an individual metabolite, with its position along the x-axis indicating the magnitude and direction of fold change: left of zero denotes higher abundance in MGUS, right of zero denotes higher abundance in MM.

Metabolic rewiring accompanies the evolution from MGUS to MM, yet how these alterations partition between the intracellular and extracellular bone marrow micro-environment is unknown^26,27^. To capture both dimensions, we combined high-resolution MALDI-FT-ICR imaging of FFPE biopsies across the entire bone marrow region with the matched bone marrow plasma LC-MS metabolite profiling. A total of 114 metabolites were quantified in both matrices, providing a shared analytical denominator (Fig. 5A, **left**).

Plotting the MM-to-MGUS log_2_-fold change (log_2_FC) in tissue (y-axis) against plasma (x-axis) stratified each metabolite into eight directional classes (Fig. 5A, **right**). Concordant shifts were prominent: 17 metabolites rose in both compartments (“Both up”) and five declined (“Both down”). The “Both up” quadrant was dominated by the kynurenine arm of tryptophan catabolism—3-hydroxy-kynurenine and kynurenate—as well as cytidine monophosphate (CMP), highlighting a programme that is simultaneously imprinted across the entire bone marrow of MM and their corresponding bone marrow plasma.

Compartment-restricted changes, whether only in the bone marrow tissue or only in the bone marrow plasma, provided additional biological resolution. Twenty-two metabolites increased only in tissue, including arachidonic acid and unmetabolised tryptophan, pointing to intrinsic or intracellular eicosanoid signalling and substrate accumulation. Conversely, eight metabolites were elevated solely in bone marrow plasma, predominantly long-chain acyl-carnitines, consistent with systemic lipid mobilisation. Offset behaviour (opposite directions) was comparatively rare but informative: 2′-deoxyuridine accumulated in tissue while falling in bone marrow plasma (tissue↑/plasma↓), suggesting local nucleotide salvage, whereas L-cystathionine decreased in tissue but rose in plasma (tissue↓/plasma↑), hinting at differential trans-sulphuration flux. Hypoxanthine, a purine-degradation product, was the most prominent tissue-specific decrease, implying enhanced nucleotide turnover within the malignant niche. Together, these data define a compartment-shared metabolic core with matrix-specific features.

To further dissect the pathway context of these directional classes, a Sankey diagram (Fig. 5B) mapped how each group of metabolites funnels into six major metabolic super-pathways. Notably, nucleotide metabolism dominated the “Both up” category, while amino-acid and lipid metabolism prevailed among tissue- and plasma-specific increases, highlighting functional divergence in metabolic reprogramming between intracellular and extracellular compartments^28^.

Beeswarm plots of all shared metabolites (Fig. 5C, **left**) showed that lipid and amino-acid super-pathways contained the widest log_2_FC distributions, while nucleotide species tended to decrease in both compartments. Extending the analysis to all tissue and bone marrow plasma metabolites (Fig. 5C, **centre and right**) confirmed a broader dynamic range in tissue and highlighted pathway-specific biases: nucleotides and carbohydrates skewed towards MM in tissue, whereas amino-acid and lipid metabolites contributed most of the bone marrow plasma signal.

We next asked whether these tissue-resolved and systemic signatures distinguish higher-risk MGUS-like marrows (e.g., SBPmm) from true MGUS cases.

### Metabolic profiling distinguishes progressive MGUS and reveals precursor heterogeneity

Applying the above framework to MGUS revealed pronounced precursor heterogeneity and uncovered subsets whose tissue metabolomes already resembled MM, even when the extracellular bone marrow plasma remained non-informative.

**Figure 6:**
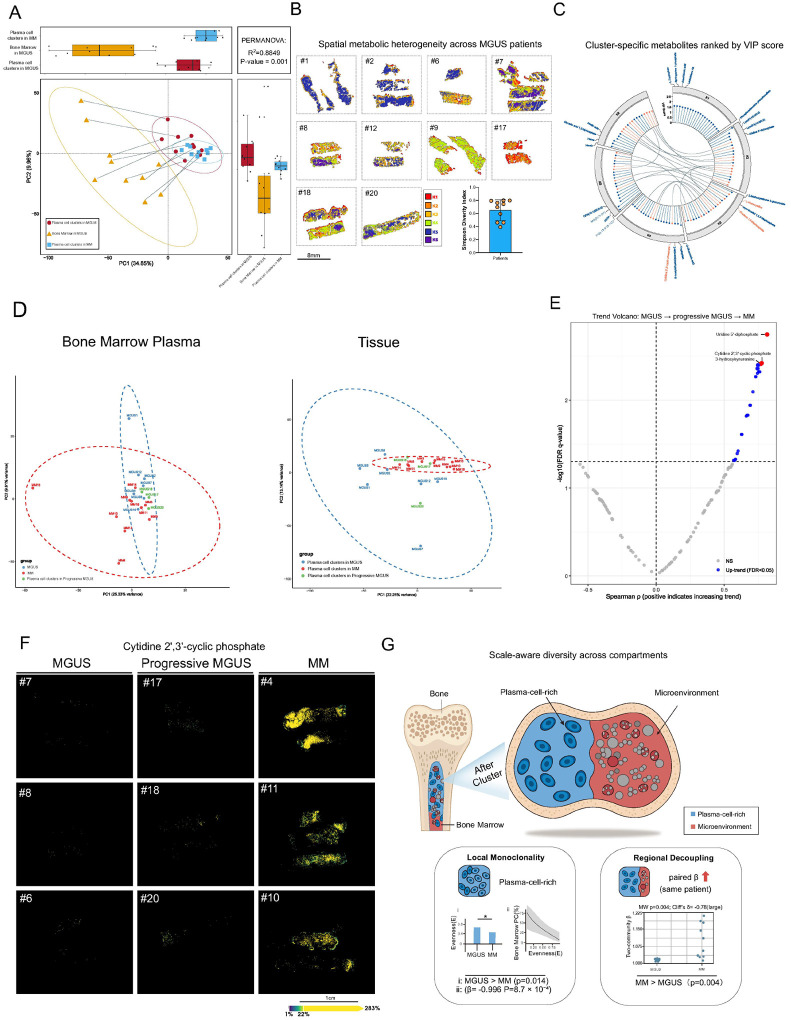
Metabolic landscape differentiating MGUS and MM progression. **A.** Principal component analysis (PCA) of metabolite profiles from three regions: Plasma cell clusters in MGUS (red), entire MGUS regions from MGUS patients (yellow), and Plasma cell clusters in MM (blue). Marginal boxplots show distributions of PC1 and PC2 scores, respectively. Group separations were evaluated using PERMANOVA (R^2^ = 0.8849, *p* = 0.001). **B.** Spatial heterogeneity within MGUS patients analyzed by Bisecting K-means clustering (K = 6). Different clusters (K1–K6) are indicated by distinct colors. Simpson’s diversity index across patients (right bottom panel) quantifies heterogeneity based on cluster distribution and proportions per patient. **C.** Circular plot illustrating the top 20 VIP-score metabolites (length of lollipop) for each of the six K-clusters. Metabolite names are labeled only for the top 4 metabolites per cluster. Orange lines indicate metabolites shared across clusters, while blue represents cluster-specific metabolites. Connections highlight shared metabolites among clusters. **D.** Principal component analysis (PCA) of metabolite profiles in two matrices. Left (Plasma): PCA computed from 823 plasma metabolites per patient. Right (Tissue): PCA computed from 3,005 metabolites quantified in MSI-clustered malignant plasma cell regions. Points indicate non-progressive MGUS, progressive MGUS, and MM (colors as in the panel keys); dashed ellipses denote 95% confidence intervals. In the tissue PCA, two progressive MGUS cases cluster with the MM group. **E.** Trend volcano plot highlighting metabolites showing monotonic increasing trends from non-progressive MGUS through progressive MGUS to MM, based on Spearman correlation analysis (*q* < 0.05). Red dots denote the top three metabolites significantly upregulated across progression stages. **F.** Representative mass spectrometry imaging of one highlighted metabolite (Cytidine 2’,3’-cyclic phosphate, *m/z* 304.03376) from (E), comparing its spatial distribution across non-progressive MGUS, progressive MGUS, and MM patients. **G.** Top, SCiLS Lab-based segmentation of each bone-marrow section into plasma-cell–rich and microenvironment compartments used for diversity metrics. Left (Local monoclonality): Evenness within the plasma-cell–rich compartment, evenness based on the Hill number with *q*=2 (*E=*^*2*^*D/K*_*eff*_) is lower in MM than MGUS (two-sided Mann–Whitney U test, *p* =0.014); evenness is inversely associated with marrow plasma-cell percentage (OLS on logit-transformed scales, slope *β*=−0.996, HC3 *p*=8.7×10^−4^; fit shown with 95% CI**).** Right (Regional decoupling): Patient-paired two-community *β* diversity (Hill *q*=2; *β*=^2^*D*_*γ*_*/*^*2*^*D*_*α, w*_, where ^2^*D*_*α, w*_ is the pixel/count-weighted mean of the plasma-cell–rich and microenvironment ^2^*D* within each patient, is higher in MM than in MGUS (two-sided Mann–Whitney U test, row p=0.004; Cliff’s *δ* (MGUS vs MM) = −0.78, large, indicating higher values in MM).

Principal-component analysis (PCA) of all detected metabolites demonstrated marked metabolic heterogeneity among MGUS bone-marrow samples (Fig. 6A). Whole-marrow regions from MGUS patients (yellow triangles), encompassing both plasma cell and non–plasma cell compartments, were widely dispersed across the first two principal components. In contrast, plasma-cell–enriched regions identified by unsupervised clustering formed substantially tighter groupings—red circles for MGUS and blue squares for MM—indicating that data-driven segmentation isolates metabolically coherent plasma-cell niches from an otherwise diffuse precursor landscape. Consistently, PERMANOVA confirmed that these region types were metabolically distinct (R2 = 0.885, p = 0.001). Thus, although MGUS bone marrow is heterogeneous at the whole-tissue level, it contains discrete plasma-cell niches with homogeneous metabolic signatures, some of which converge toward an MM-like state.

Restricting the analysis to MGUS samples, Fig. 6B further illustrates substantial intraprecursor heterogeneity. Six spatial clusters (K1–K6) intermingled differently across patients, and Simpson’s diversity indices varied widely (≈ 0.4–0.8), indicating that the MGUS niche is not metabolically uniform. Together, Figs. 6A and 6B establish MGUS as a metabolically diverse precursor state, consistent with reported heterogeneity in immune surveillance and clonal biology that may contribute to divergent risks of progression^29^.

To further delineate this heterogeneity, we performed cluster-level analysis of endogenous metabolites. Six distinct metabolic regions were identified, and metabolites were ranked within each cluster by variable importance in projection (VIP) scores. Figure 6C shows the top 20 metabolites per cluster, with the top four labeled. Several metabolites recurred as high-ranking discriminators across multiple clusters (highlighted in orange), indicating shared metabolic features, whereas others were cluster-specific, reflecting localized metabolic specialization. This analysis highlights the coexistence of common and region-restricted metabolic programs within MGUS/MM bone marrow.

We next examined three SBPmm patients within the MGUS cohort who subsequently progressed to MM. In PCA of bone marrow plasma metabolomes (Fig. 6D, left), these cases overlapped with the MGUS distribution and did not consistently shift toward MM. In contrast, PCA of MSI-defined plasma-cell–enriched tissue regions (Fig. 6D, right) revealed clear stage-related structure: MGUS17 and MGUS18 clustered within the MM ellipse, whereas MGUS20 remained closer to the MGUS group. Thus, spatially resolved tissue metabolomics captured MM-like metabolic features in clonal plasma cells of a subset of MGUS-like marrows that were not evident from bone marrow plasma alone.

Notably, the time to progression among these SBPmm cases varied substantially, ranging from approximately 24 months (MGUS17) to 7 months (MGUS18) and 2 months (MGUS20). This clinical variability was mirrored by differences in metabolic organization. MGUS17, the slowest progressor, exhibited the lowest Simpson index (0.59), whereas MGUS18 and MGUS20 showed higher indices (0.80 and 0.76), consistent with stronger metabolic dominance. These observations suggest that early emergence of a dominant metabolic program—rather than increased heterogeneity per se—may be associated with more rapid progression from MGUS to MM.

In addition to these multivariate patterns, we identified metabolites that increased monotonically across true MGUS, MGUS-like SBPmm, and MM (Fig. 6E). Among the top five were cytidine 2′,3′-cyclic phosphate and 3-HK. Cytidine 2′,3′-cyclic phosphate showed a stepwise increase from non-progressive MGUS to progressive MGUS and MM, indicating that some myeloma-associated metabolic alterations are already present at the precursor stage. Similarly, rising 3-HK levels underscore activation of the kynurenine pathway during progression. Although mechanistic dissection is beyond the scope of this analysis, these consistent trajectories indicate that progressive MGUS harbors metabolic features bridging precursor and malignant states.

To assess how this heterogeneity is organized spatially, we segmented each section into plasma-cell–rich and microenvironmental compartments and quantified diversity using Hill numbers (q = 2) (Fig. 6G). Here, “evenness” (q = 2) quantifies how balanced metabolite abundances are within a region; lower values indicate dominance by a few features and should not be conflated with spatial homogeneity across compartments. This framework links within-region evenness, between-region differentiation, and whole-section heterogeneity in a single system. Within the plasma-cell-rich compartment of each bone marrow sample, evenness was lower in MM than in MGUS (p = 0.014; Fig. 6G, left, panel i). The evenness also showed a strong inverse association with marrow plasma-cell percentage (slope β = −0.996, HC3 p = 8.7 × 10–4; Fig. 6G, left, panel ii). These findings indicate thatregions with higher plasma cell infiltration exhibit more metabolically dominated and less evenly distributed metabolite profiles — consistent with local metabolic simplification during progression.. We next compared the two compartments, plasma cell rich region vs. non-plasma cell region within each patient. The paired two-community β diversity increased in MM (p = 0.004; Cliff’s δ = −0.78, large; Fig. 6G, right). Thus, the metabolic composition of the plasma cell core and the surrounding microenvironment in the bone marrow diverges during progression from MGUS to MM. The microenvironment does not simply mirror the plasma cell-rich region; rather, it drifts away from it.

Together, Fig. 6G closes the loop opened in Fig. 6A–F. Early, MGUS appears heterogeneous in the whole marrow but contains tight, metabolically coherent plasma-cell niches. As disease advances, the plasma cell-rich niche becomes more monoclonal and the microenvironment decouples from it^30^. This scale-aware view reconciles local clonality with global diversity and points to niche separation as a key ecological feature of MGUS-to-MM transition.

## Discussion

By integrating high-resolution MALDI-FT-ICR imaging of FFPE bone-marrow biopsies with matched bone marrow plasma metabolite profiling by LC–MS in MGUS and MM patients, we delineate how coordinated changes in local clonal plasma cell metabolism and the systemic metabolic milieu accompany progression from precursor to malignant states. Across compartments, we identify a conserved metabolic core characterized by enhanced tryptophan–kynurenine flux and rewired nucleotide metabolism, together with compartment-specific lipid remodeling. Spatial clustering isolates plasma-cell niches, reveals marked precursor heterogeneity, and identifies MGUS subsets whose tissue metabolomes already resemble MM—even when bone marrow plasma profiles remain non-informative. Coupling analyses further link proliferative activity to stage-dependent shifts in lipid mediators and nucleotide biosynthesis, providing a spatial–systemic framework for understanding MGUS-to-MM evolution and nominating metabolite signatures for future risk-oriented stratification.

A central feature of progression is coordinated reprogramming of the tryptophan–kynurenine axis and nucleotide metabolism, with stage-dependent engagement of lipid signaling. MM patients exhibited depleted tryptophan and elevated 3-HK relative to MGUS (Fig. 3), consistent with prior studies of bone marrow fluid and plasma^11^. The increase in 3-HK reflects enhanced flux through the kynurenine pathway, likely driven by upregulated IDO1 activity^31^. Rather than acting in isolation, this pathway-level shift integrates with proliferation: in MGUS, proliferative activity associates primarily with carbohydrate and UDP-sugar metabolism, whereas in MM it aligns with bioactive lipids (sphingosine-1-phosphate and arachidonic acid) and increased purine and pyrimidine biosynthesis (Fig. 4)^20,25,26^. Together, these changes position immune-modulatory tryptophan catabolism within a broader anabolic program that scales with disease stage and proliferative demand.

Beyond individual pathways, our study addresses a key limitation of prior metabolomic analyses in plasma cell disorders, which have typically examined either bone marrow microenvironmental compartments or peripheral circulation in isolation^11,13,32^. By profiling metabolites in intact bone marrow tissue alongside matched bone marrow plasma from the same individuals (Fig. 5), we demonstrate that some metabolic alterations are conserved across compartments, whereas others are compartment restricted. For example, arachidonic acid and unmetabolised substrates such as tryptophan accumulated locally within MM marrow, whereas long-chain acyl-carnitines increased predominantly in bone marrow plasma, consistent with systemic lipid mobilization^33^ and cancer-associated catabolism^34^. Metabolites displaying opposite directional changes between tissue and plasma (e.g., 2′-deoxyuridine) suggest localized nucleotide salvage coupled to systemic redistribution of one-carbon and amino-acid flux. These findings extend prior observations in bone marrow plasma by directly linking systemic metabolic alterations to tumor-intrinsic metabolic programs^11^. By integrating spatial and plasma metabolomics, our study provides a more comprehensive view of MGUS and MM metabolism and informs which tumor-associated metabolites may be most amenable to evaluation as circulating biomarkers or therapeutic targets.

A key biological insight from our spatial analysis is the pronounced metabolic heterogeneity within MGUS and its clinical relevance. Unsupervised clustering of bone-marrow metabolite images (Fig. 6) demonstrates that MGUS is not metabolically uniform: some cases harbor plasma-cell tissue metabolomes that already resemble MM, whereas others retain indolent-like profiles. Importantly, this tissue-resolved stratification identified MGUS-like bone marrows from SBPmm patients who later progressed to MM, distinguishing them from true MGUS cases that remained progression-free, whereas matched bone marrow plasma metabolomes were less discriminative (Fig. 6). Given the variability in MGUS progression risk and the limitations of current clinical and molecular risk models^35^, these findings suggest that spatial metabolomics provides an orthogonal layer of risk assessment grounded in tumor metabolism. Similar principles of tissue-based metabolic subtyping have proven prognostically informative in solid tumors^36^, and our data extend this concept to a pre-malignant hematologic condition. Consistent with evolutionary models derived from single-cell studies^37^ and recent reviews^38^, our results indicate that MGUS progression can be captured at the metabolic level through spatially resolved tissue profiling.

These observations prompted us to examine how heterogeneity is organized across spatial scales. Using a scale-aware diversity framework based on Hill numbers (q = 2) (Fig. 6G), we identify two complementary features of progression. First, local metabolic dominance: evenness within plasma-cell–rich regions was reduced in MM relative to MGUS and declined with increasing marrow plasma-cell burden, linking tumor expansion to a narrowing of the active metabolic repertoire. Second, regional decoupling: paired β diversity between plasma-cell cores and surrounding microenvironment increased in MM, indicating progressive metabolic divergence between tumor and niche compartments. These findings parallel spatial-immunology studies showing compartmentalization of immune architecture during MGUS-to-MM transition^39^.

Together, these scale-dependent metrics suggest that disease progression reflects ecological reorganization—local clonal dominance combined with regional niche separation—rather than simple accumulation of intratumoral complexity. Metrics capturing between-compartment divergence may therefore complement existing staging approaches and highlight tumor–microenvironment interactions as potential intervention points, consistent with emerging ecological and systems-level models of cancer progression^38^.

A major strength of our study lies in the first systematic application of MALDI–FT-ICR spatial metabolomics to diagnostic FFPE bone marrow biopsies. While prior studies have largely focused on fresh or frozen tissues, we demonstrate the feasibility and robustness of this approach on routinely archived material. High-resolution spatial mapping not only enables characterization of individual plasma cell niches but also quantifies metabolic interactions with the surrounding microenvironment. This methodological advance expands the potential for retrospective analyses across large clinical cohorts and provides a directly translatable strategy for integrating spatial metabolomics into diagnostic and prognostic workflows in plasma cell disorders.

In summary, our study demonstrates the power of combining spatial and systemic metabolomics to unravel disease-associated metabolism and clarifies how local tumor programs and systemic remodeling jointly contribute to myeloma pathogenesis. These insights lay a foundation for metabolite-based biomarkers and metabolic interventions aimed at intercepting MGUS-to-MM progression. Nevertheless, interpretation is tempered by a modest cohort, the largely cross-sectional design, and the absence of genomic and immune-composition integration. Validation in larger, prospectively accrued cohorts—including SMM—and longitudinal sampling will be required to determine whether MM-like tissue profiles in MGUS anticipate progression. Future integration of spatial metabolomics with cytogenetics and single-cell phenotyping should further determine whether the observed regional decoupling reflects tumor-intrinsic programs or microenvironmental remodeling.

## Supplementary Material

Supplementary Files

This is a list of supplementary files associated with this preprint. Click to download.

- SupplementalMethods.docx

## Figures and Tables

**Table 1:** Baseline characteristics of the study cohorts. Patients with MGUS (n = 10) and multiple myeloma (MM; n = 10) undergoing standard-of-care bone marrow biopsy/aspiration at Mayo Clinic were included. Diagnoses followed International Myeloma Working Group criteria. The study was approved by the Mayo Clinic IRB (15–005140) and conducted in accordance with the Declaration of Helsinki. Values are median (range) or n (%). BMPCs, bone marrow plasma cells; S-phase, proliferative fraction by clinical flow cytometry; SBP, solitary bone plasmacytoma (concurrent at diagnosis).

All patients(N = 20)		
Characteristic	MGUS(N = 10)	MM(N = 10)
**Median Age (Range)**	67 (54–89)	68 (46–81)
**Gender (Male %)**	6 (60%)	6 (60%)
**BMPCs% (Range)**	5 (3–7)	70 (50–90)
**S-phase% (Range)**	0.5 (0.2–3.3)	0.7 (< 0.1–8.7)
**Concurrent SBP**	3 (30%)	N/A

## Data Availability

The datasets generated or analyzed during the current study are available on reasonable request from the corresponding authors. Custom analysis scripts are publicly available at GitHub (https://github.com/guoxingzhanggx-del/spatial-metabolomics-mm.git). Additional details about materials and methods are provided in the Supplemental Methods.

## References

[1] Dimopoulos MA, Terpos E, Boccadoro M, Moreau P, Mateos MV, Zweegman S, EHA-EMN Evidence-Based Guidelines for diagnosis, treatment and follow-up of patients with multiple myeloma. Nat Rev Clin Oncol, (2025). 10.1038/s41571-025-01041-x. 40624367

[2] Sun F, Cheng Y, Ying J, Mery D, Al Hadidi S, Wanchai V, A gene signature can predict risk of MGUS progressing to multiple myeloma. J Hematol Oncol 16, 70 (2023). 10.1186/s13045-023-01472-y. 37386588 PMC10308756

[3] van de Donk N, Pawlyn C, Yong KL. Multiple myeloma. Lancet 397, 410–27 (2021). 10.1016/s0140-6736(21)00135-5. 33516340

[4] Zhang F, Ma Y, Li D, Wei J, Chen K, Zhang E, Cancer associated fibroblasts and metabolic reprogramming: unraveling the intricate crosstalk in tumor evolution. J Hematol Oncol 17, 80 (2024). 10.1186/s13045-024-01600-2. 39223656 PMC11367794

[5] Hu T, Liu CH, Lei M, Zeng Q, Li L, Tang H, Metabolic regulation of the immune system in health and diseases: mechanisms and interventions. Signal Transduct Target Ther 9, 268 (2024). 10.1038/s41392-024-01954-6. 39379377 PMC11461632

[6] Ramberger E, Sapozhnikova V, Ng YLD, Dolnik A, Ziehm M, Popp O, The proteogenomic landscape of multiple myeloma reveals insights into disease biology and therapeutic opportunities. Nat Cancer 5, 1267–84 (2024). 10.1038/s43018-024-00784-3. 38942927 PMC11358022

[7] Xiao Y, Li Y, Zhao H. Spatiotemporal metabolomic approaches to the cancer-immunity panorama: a methodological perspective. Mol Cancer 23, 202 (2024). 10.1186/s12943-024-02113-9. 39294747 PMC11409752

[8] Liu X, Peng T, Xu M, Lin S, Hu B, Chu T, Spatial multi-omics: deciphering technological landscape of integration of multi-omics and its applications. J Hematol Oncol 17, 72 (2024). 10.1186/s13045-024-01596-9. 39182134 PMC11344930

[9] Desantis V, Andriano A, Düking T, Hartwig O, Ingravallo G, Biondo M, Spatial imaging unlocks the potential of charting multiple myeloma and extramedullary disease. J Hematol Oncol 18, 47 (2025). 10.1186/s13045-025-01699-x. 40269964 PMC12020055

[10] Cui H, Tejada-Lapuerta A, Brbić M, Saez-Rodriguez J, Cristea S, Goodarzi H, Towards multimodal foundation models in molecular cell biology. Nature 640, 623–33 (2025). 10.1038/s41586-025-08710-y. 40240854

[11] Gonsalves WI, Broniowska K, Jessen E, Petterson XM, Bush AG, Gransee J, Metabolomic and Lipidomic Profiling of Bone Marrow Plasma Differentiates Patients with Monoclonal Gammopathy of Undetermined Significance from Multiple Myeloma. Sci Rep 10, 10250 (2020). 10.1038/s41598-020-67105-3. 32581232 PMC7314797

[12] Ray A, Song Y, Du T, Tai YT, Chauhan D, Anderson KC. Targeting tryptophan catabolic kynurenine pathway enhances antitumor immunity and cytotoxicity in multiple myeloma. Leukemia 34, 567–77 (2020). 10.1038/s41375-019-0558-x. 31462737 PMC7132142

[13] Ludwig C, Williams DS, Bartlett DB, Essex SJ, McNee G, Allwood JW, Alterations in bone marrow metabolism are an early and consistent feature during the development of MGUS and multiple myeloma. Blood Cancer J 5, e359 (2015). 10.1038/bcj.2015.85. 26473531 PMC4635194

[14] Bhattacharjee S, Saha B, Saha S. Symptom-based drug prediction of lifestyle-related chronic diseases using unsupervised machine learning techniques. Comput Biol Med 174, 108413 (2024). 10.1016/j.compbiomed.2024.108413. 38608323

[15] Xiong D, Wang Q, Wang WM, Sun ZJ. Tuning cellular metabolism for cancer virotherapy. Cancer Lett 592, 216924 (2024). 10.1016/j.canlet.2024.216924. 38718886

[16] Borsa M, Obba S, Richter FC, Zhang H, Riffelmacher T, Carrelha J, Autophagy preserves hematopoietic stem cells by restraining MTORC1-mediated cellular anabolism. Autophagy 20, 45–57 (2024). 10.1080/15548627.2023.2247310. 37614038 PMC10761185

[17] Asano A, Ri M, Masaki A, Maeda Y, Tachita T, Hirade K, Aberrant tryptophan metabolism leads to unfavorable outcomes in lenalidomide-treated myeloma patients. Hematol Oncol 41, 424–33 (2023). 10.1002/hon.3108. 36426594

[18] Morelli E, Ribeiro CF, Rodrigues SD, Gao C, Socciarelli F, Maisano D, Targeting Acetyl-CoA Carboxylase Suppresses De Novo Lipogenesis and Tumor Cell Growth in Multiple Myeloma. Clin Cancer Res 31, 1975–87 (2025). 10.1158/1078-0432.Ccr-24-2000. 40053701 PMC12081190

[19] Zanwar S, Jevremovic D, Kapoor P, Olteanu H, Buadi F, Horna P, Clonal plasma cell proportion in the synthetic phase identifies a unique high-risk cohort in multiple myeloma. Blood Cancer J 15, 20 (2025). 10.1038/s41408-025-01232-w. 39966346 PMC11836398

[20] Leng H, Simon AK, Horwood NJ. Blocking glycosphingolipid production alters autophagy in osteoclasts and improves myeloma bone disease. Autophagy 20, 930–2 (2024). 10.1080/15548627.2023.2208931. 37771244 PMC11062377

[21] Leng H, Zhang H, Li L, Zhang S, Wang Y, Chavda SJ, Modulating glycosphingolipid metabolism and autophagy improves outcomes in pre-clinical models of myeloma bone disease. Nat Commun 13, 7868 (2022). 10.1038/s41467-022-35358-3. 36550101 PMC9780346

[22] Tran DH, Kim D, Kesavan R, Brown H, Dey T, Soflaee MH, De novo and salvage purine synthesis pathways across tissues and tumors. Cell 187, 3602–18.e20 (2024). 10.1016/j.cell.2024.05.011. 38823389 PMC11246224

[23] Mullen NJ, Singh PK. Nucleotide metabolism: a pan-cancer metabolic dependency. Nat Rev Cancer 23, 275–94 (2023). 10.1038/s41568-023-00557-7. 36973407 PMC10041518

[24] Pfeiffer C, Grandits AM, Asnagli H, Schneller A, Huber J, Zojer N, CTPS1 is a novel therapeutic target in multiple myeloma which synergizes with inhibition of CHEK1, ATR or WEE1. Leukemia 38, 181 – 92 (2024). 10.1038/s41375-023-02071-z. 37898670

[25] Nair R, Vu AH, Freer AK, Bhatia KS, Wang D, Savani MR, Heme promotes venetoclax resistance in multiple myeloma through MEK-ERK signaling and purine biosynthesis. Blood 145, 732–47 (2025). 10.1182/blood.2024025690. 39693611 PMC12060166

[26] Oudaert I, Van der Vreken A, Maes A, De Bruyne E, De Veirman K, Vanderkerken K, Metabolic cross-talk within the bone marrow milieu: focus on multiple myeloma. Exp Hematol Oncol 11, 49 (2022). 10.1186/s40164-022-00303-z. 36050788 PMC9438316

[27] Panaroni C, Fulzele K, Mori T, Siu KT, Onyewadume C, Maebius A, Multiple myeloma cells induce lipolysis in adipocytes and uptake fatty acids through fatty acid transporter proteins. Blood 139, 876–88 (2022). 10.1182/blood.2021013832. 34662370 PMC8832479

[28] Navarro F, Lozano T, Fuentes-García A, Sánchez-Moreno I, Larrayoz M, Justicia P, Reprogramming glutamine metabolism enhances BCMA-CART cell fitness and therapeutic efficacy in multiple myeloma. Blood, (2025). 10.1182/blood.2024027496. PMC12824695 40971494

[29] DhodapkarMV, PaivaB. Immune alterations in myeloma evolution and outcomes: quo vadis? Blood 146, 1041–50 (2025). 10.1182/blood.2024026227.40623045

[30] Chen M, Jiang J, Hou J. Single-cell technologies in multiple myeloma: new insights into disease pathogenesis and translational implications. Biomark Res 11, 55 (2023). 10.1186/s40364-023-00502-8. 37259170 PMC10234006

[31] Stone TW, Williams RO. Modulation of T cells by tryptophan metabolites in the kynurenine pathway. Trends Pharmacol Sci 44, 442–56 (2023). 10.1016/j.tips.2023.04.006. 37248103

[32] Dunphy K, Bazou D, Henry M, Meleady P, Miettinen JJ, Heckman CA, Proteomic and Metabolomic Analysis of Bone Marrow and Plasma from Patients with Extramedullary Multiple Myeloma Identifies Distinct Protein and Metabolite Signatures. Cancers (Basel) 15, (2023). 10.3390/cancers15153764. PMC10417544 37568580

[33] Simcox J, Geoghegan G, Maschek JA, Bensard CL, Pasquali M, Miao R, Global Analysis of Plasma Lipids Identifies Liver-Derived Acylcarnitines as a Fuel Source for Brown Fat Thermogenesis. Cell Metab 26, 509 – 22.e6 (2017). 10.1016/j.cmet.2017.08.006. 28877455 PMC5658052

[34] Catanese S, Beuchel CF, Sawall T, Lordick F, Brauer R, Scholz M, Biomarkers related to fatty acid oxidative capacity are predictive for continued weight loss in cachectic cancer patients. J Cachexia Sarcopenia Muscle 12, 2101–10 (2021). 10.1002/jcsm.12817. 34636159 PMC8718041

[35] Liu Y, Parks AL. Diagnosis and Management of Monoclonal Gammopathy of Undetermined Significance: A Review. JAMA Intern Med 185, 450–6 (2025). 10.1001/jamainternmed.2024.8124. 39960681 PMC11975479

[36] Wang J, Sun N, Kunzke T, Shen J, Zens P, Prade VM, Spatial metabolomics identifies distinct tumor-specific and stroma-specific subtypes in patients with lung squamous cell carcinoma. NPJ Precis Oncol 7, 114 (2023). 10.1038/s41698-023-00434-4. 37919427 PMC10622419

[37] Dang M, Wang R, Lee HC, Patel KK, Becnel MR, Han G, Single cell clonotypic and transcriptional evolution of multiple myeloma precursor disease. Cancer Cell 41, 1032–47.e4 (2023). 10.1016/j.ccell.2023.05.007. 37311413 PMC10317474

[38] Dutta AK, Alberge JB, Sklavenitis-Pistofidis R, Lightbody ED, Getz G, Ghobrial IM. Single-cell profiling of tumour evolution in multiple myeloma - opportunities for precision medicine. Nat Rev Clin Oncol 19, 223–36 (2022). 10.1038/s41571-021-00593-y. 35017721

[39] Robinson MH, Villa NY, Jaye DL, Nooka AK, Duffy A, McCachren SS, Regulation of antigen-specific T cell infiltration and spatial architecture in multiple myeloma and premalignancy. J Clin Invest 133, (2023). 10.1172/jci167629. PMC10378152 37526080

